# Effects of a Web-Based Patient Portal on Patient Satisfaction and Missed Appointment Rates: Survey Study

**DOI:** 10.2196/17955

**Published:** 2020-05-19

**Authors:** Timothy A D Graham, Samina Ali, Melita Avdagovska, Mark Ballermann

**Affiliations:** 1 School of Public Health University of Alberta Edmonton, AB Canada; 2 Alberta Health Services Edmonton, AB Canada; 3 Women and Children's Health Research Institute University of Alberta Edmonton, AB Canada; 4 Department of Critical Care Medicine University of Alberta Edmonton, AB Canada

**Keywords:** patient portal, patient satisfaction

## Abstract

**Background:**

Although electronic medical record (EMR)-tethered patient portals are common in other countries, they are still emerging in Canada.

**Objective:**

We aimed to report user satisfaction and the effects of a patient portal on medical appointment attendance in a Canadian cohort of patients within our publicly funded health care system.

**Methods:**

Two surveys were deployed, via email, at 2 weeks and 6 months following the first recorded patient portal access. Database audits of visit attendance were used to supplement and cross reference survey data.

**Results:**

Between January 2016 and July 2018, 4296 patients accessed the patient portal. During the study, 28% (957/3421) consented patient portal users responded to one or more semistructured electronic surveys. Of respondents, 93% (891/957) reported that the patient portal was easy to use, 51% (492/975) reported it saved time when scheduling an appointment, and 40% (382/957) reported that they had to repeat themselves less during appointments. Respondents reported patient portal–related changes in health system use, with 48% (462/957) reporting avoiding a clinic visit and 2.7% (26/957) avoiding an emergency department visit. Across 19,968 visits in clinics where the patient portal was introduced, missed appointments were recorded in 9.5% (858/9021) of non–patient portal user visits, compared with 4.5% (493/9021) for patient portal users, representing a 53% relative reduction in no-show rates.

**Conclusions:**

Early experience with an EMR-tethered patient portal showed strong reports of positive patient experience, a self-reported decrease in health system use, and a measured decrease in missed appointment rates. Implications on the expanded use of patient portals requires more quantitative and qualitative study in Canada.

## Introduction

Canadians desire electronic access to their health information and have an expectation of managing their health system interactions digitally [1]. Increasingly, that access is provided through electronic medical record (EMR)-tethered and standalone web-based patient portals [2-12]. Patient portals can deliver digital access to secure messaging, health information (eg, test results, lists of medications and allergies), scheduling functions, and self-management of health issues such as weight, blood glucose, and blood pressure. Early studies of patient portals reported patient benefits including enhanced satisfaction, better relationships with their care providers, more efficient medication refills, and improved understanding of their health information [3,4,11,13-16]. Health system benefits include fewer missed clinic appointments (no-shows), lower postage costs related to decreased mailing of paper records to patients, and less time answering phone calls related to appointments [3,4,11,13-16]. A recent systematic review of patient portals concluded that there is an urgent need for more outcomes data and reporting on organizational and provider context, as well as implementation processes [17].

Fully interactive patient portals that share detailed health information, messaging, and scheduling capability are relatively rare in Canada, and mostly allow patients limited interaction with a single health care facility or clinic [2,18-22]. A number of provinces provide standalone portals that aggregate laboratory and diagnostic imaging results, but few employ patient portals that allow self-scheduling or health management across a large population. Alberta Health Services (AHS) had a limited production rollout of a patient portal tethered to an EMR in the Edmonton zone from 2016-2019. The objectives of our study were to examine the effects of this tethered patient portal on patient satisfaction and health system usage.

## Methods

### Design

For this study, we employed surveys and system audit logs. This combination of longitudinal semistructured user surveys and administrative data audit was used to identify user-reported outcomes triangulated with possible clinical outcomes of interest in a cohort of users attending clinics allowing access to a patient portal. The University of Alberta’s Health Research Ethics Board (Pro00066287) approved the study.

### Patient Portal Development and Adoption

The patient portal that we studied is tethered to a shared EMR known as eCLINICIAN (an AHS branding of EpicCare Ambulatory 2014 from Epic Systems), used at the time by approximately 1110 physicians, residents, and students, and approximately 6000 nurses, allied health professionals, and other staff. The adaptation of this patient portal to the AHS context took about six months, the bulk of which was spent achieving consensus (including patient, clinical, administrative, legislative, and regulatory college input) on policy decisions and functions (Textbox 1). Consensus was reached to release most high-value data in real time (ie, those data required to manage active or chronic disease), with higher stakes information (ie, results of hepatitis or biopsies) delayed by ten days (Textbox 2).

**Patient portal features.**

- Secure bidirectional messaging between patient and care team
- Display medical information (medications, problems, past history, allergies, immunizations)
- Create, select, view, and modify scheduled appointments (varies by clinic)
- View appointments and receive reminders sent to patient mobile devices
- Complete previsit questionnaires and patient-entered flowsheets
- Enter and track home data (eg, blood pressure, glucose)
- iOS and Android mobile apps
- Integration with Alberta Personal Health Portal
- Email signup process to reduce initial 2-factor login issues for patients

**Results release framework.**

**Results released in real time (hourly)**

- Hematology (complete blood count, white blood cell count), electrolytes, renal function, liver function, lipids, diabetes monitoring (glucose, HbA_1C_), coagulation profile (international normalized ratio, prothrombin time test), cardiac tests (troponin, B-type natriuretic peptide), sexually transmitted infection results, bacterial cultures

**Results released after 10-day delay (or earlier if provider does so manually)**

- Pathology reports, diagnostic imaging reports, genetic testing (newborn screening, specific diagnostic testing (eg, for angioedema, protein C deficiency, carrier testing, prenatal testing, preimplementation testing), cytogenetics, histocompatibility, serology, molecular diagnostics, HIV serology, hepatitis C serology, cancer-related markers (eg, prostate-specific antigen, carcinoembryonic antigen)

### Study Setting and Population

AHS is a provincial health system responsible for the delivery of inpatient and ambulatory care services. Edmonton is the capital city of Alberta, with a population of approximately one million. In January 2016, an EMR-tethered patient portal was introduced in the Edmonton zone, and was evaluated between January 2016 and July 2018. During this phase, the patient portal was introduced in one family practice clinic and four specialty clinics (rheumatology, inflammatory bowel disease, multiple sclerosis, and diabetes). These specialty clinics are part of a large academic health campus, accept patients initially via referral for specialty care, then follow them for their chronic disease management. Inclusion criteria for the survey study were the following: patients who had (1) visited 1 of the 5 aforementioned clinics between January 2016 and July 2018, and (2) signed up for patient portal access. A total of 3421 patients met inclusion criteria for the survey during the study period.

### Patient Survey Instrument

A novel 30-question survey instrument (Multimedia Appendix 1) was developed by members of our research team using a modified Delphi [23] approach. Four overarching themes were identified: (1) satisfaction with the patient portal, (2) utility of the patient portal, (3) impact of the patient portal, and (4) demographic characteristics. Proposed questions were refined by the research team and then pretested with 6 members of our patient portal working group. After further refinement, which included changes and clarification of wording and question structure, the survey tool was piloted and sensibility-tested with a small group of patients, leading to a final survey with 5 (satisfaction, utility) to 12 (impact) questions per theme. The survey required about 10-15 minutes to complete.

During the sign-up process for the patient portal, patients consented to be contacted via their email address, which was routinely recorded as part of the sign-up process. Patient portal users were emailed a voluntary link to the survey which contained Likert scales, multiple-choice questions and free-text questions. The free-text answers were collated and thematically coded by two authors (TG, SA). Coding discrepancies were resolved by consensus. Surveys were administered at 2-week and 6-month time points relative to each participant’s first successful access to the patient portal, as this was felt to represent an adequate period of time for participants to access and become comfortable with using the patient portal. Respondents were made aware that their responses would not impact their clinical care, that no incentives were provided, and that their responses would only be studied in collated form. Respondents were permitted to skip any question they wished.

### Appointment Attendance Records

To determine the no-show rate, the numerator of scheduled appointments recorded as no-show was divided by the denominator of total scheduled appointments. No-show rates were calculated for users with an active patient portal account at the time of the visit and compared to rates for users attending the same clinics without a patient portal account at the time of the visit. Audit log analysis included attended and no-show visit rates, basic patient demographic data, and the patient’s contact email address.

### Statistical Analysis

Categorical data (eg, sex, age) were summarized with frequency distributions. Respondent demographic characteristics were compared to the wider user population with respect to sex and age. No-show rates were compared for patient visits where a patient portal account was active at the time and patient visits in the same clinic with no active patient portal account between November 2015 (just before the patient portal was introduced) and July 2018. Each respondent’s final answer across the 3 time points to questions of a more summative nature were included for statistical testing. Statistical differences were detected at a *P* value of less than .05. Chi-square testing for each of these comparisons was conducted using R statistical software (version 3.4.3, R Core Team, R Foundation for Statistical Computing). We included users who enrolled to use the system between May 2016 and May 2018. An additional 875 new users signed up for patient portal access between May and July of 2018. To avoid biasing the estimate of the proportions of users toward the early users, these users were included in the user population but not in survey analysis.

## Results

### Demographic Characteristics

A total of 5629 electronic surveys were transmitted to 3421 patients between January 2016 and July 2018. The final administration of the survey was completed in May 2018 to allow users 2 months to respond to the survey. Overall, 957 patients responded 1916 times, and 28% (957/3421) of users receiving an invitation to participate replied to at least one survey. In addition to accessing their own health information, patient portal users reported using the patient portal for their children (36/957, 4%) or their spouse (131/957, 16%). Of 174,298 login sessions, 70% (122,648/174,298) were from a computer browser, 20% (34,205/174,298) were from an iPhone, and 10% (17,445) were from an Android device.

Table 1 describes user and respondent demographics and comfort with computer use; the majority of respondents (834/957, 87%) reported being either comfortable or completely comfortable using computers). 

**Table 1:** Patient portal user and respondent demographics.

Characteristics		Respondent frequency, n (%), (N=957)	User frequency, n (%), (N=4296)	*P* value
**Sex**				>.99
	Female	537 (56.1)	2347 (59.3)	
	Male	378 (39.5)	1749 (40.7)	
	No answer	43 (4.3)	N/A^a^	
**Age (years)**				.22
	<18	0 (0)	73 (1.7)	
	18-29	64 (6.7)	719 (16.7)	
	30-39	111 (11.6)	870 (20.3)	
	40-49	132 (13.8)	742 (17.3)	
	50-59	249 (26.0)	893 (20.8)	
	60-69	200 (20.9)	630 (14.7)	
	≥70	105 (11.0)	369 (8.6)	
	No answer	96 (10.0)	N/A	
**Comfort with computers**				
	Completely comfortable	297 (31.0)	N/A	
	Comfortable	537 (56.1)	N/A	
	Neutral	297 (31.0)	N/A	
	Uncomfortable	80 (8.4)	N/A	
	Completely uncomfortable	2 (0.2)	N/A	
	No answer	34 (3.6)	N/A	

^a^N/A: Not applicable.

### Satisfaction, Impact, and Utility

Patient portal users reported a high degree of usability and general satisfaction: 93% (891/957) of respondents felt the patient portal was easy to use, 83% (794/957) said it made communication more convenient, and 75% (716/957) indicated it saved time when scheduling an appointment.

Among survey respondents, 48% (460/957) stated the patient portal had helped them avoid a clinic visit. In February 2016, we added an additional question regarding emergency department or urgent care visit avoidance, and of users who answered this, 14% (26/188) answered that the patient portal allowed them to avoid such a visit.

There were 4 free-text questions available based on the themes of requested improvements, preferred features, how the patient portal helped avoid a health care visit (if applicable), and self-reported cost savings related to the patient portal (Table 2). Over two-thirds of respondents identified that access to laboratory and diagnostic imaging results was the most preferred feature of the patient portal. The most frequently reported concerns were related to a cumbersome sign-in process and a desire for more explanation of results. It should be noted that the patient portal was launched with an authentication method that was acknowledged to be overly complex at the time of implementation but was required for local technical and security reasons. Less common requests included a wider availability of data, including physician notes on the chart, an explanation of the meaning of the results, and the timeliness of the reports in the system.

**Table 2:** Respondents’ view on the patient portal.

Participant responses^a^		Frequency, n (%)
**Requested improvements (n=558)**		
	Less cumbersome login authentication	144 (25.8)
	More data types and wider spread availability	143 (25.6)
	Like it/love it in current state	94 (16.8)
	Better instructions/explanation of results	47 (8.4)
	Reports delayed or incomplete	43 (7.7)
	Other^b^	108 (19.4)
**Most preferred features (n=173)**		
	Lab and diagnostic imaging results	119 (68.8)
	Feeling of health empowerment	23 (13.3)
	Scheduling features	21 (12.1)
	Communication features	14 (8.1)
	Other^c^	4 (2.3)
**Factors resulting in self-reported avoidance of an emergency department/urgent care center visit (n=129)**		
	Access to results meant a visit could be avoided	76 (58.9)
	Chronic disease–specific management	22 (17.1)
	Question answered via secure message^d^	21 (16.3)
	Other^e^	50 (38.8)
**Reported cost savings by avoiding a health care visit (n=112)**		
	General convenience and less waiting in clinic	43 (38.4)
	Not paying for parking and gas	25 (22.3)
	Overall improved efficiency for the health system	19 (17.0)
	Not paying for meals/hotels	10 (8.9)
	Other^f^	2 (1.8)

^a^There was more than one answer permitted per category.

^b^Other included general usability and results management (n=41), adding and updating health information (n=20), view diagnostic imaging images (n=14), secure messaging and notifications (n=14), patients able to upload pictures and documents (n=11), and better phone/tablet experience (n=8).

^c^Other included access to health information decreases anxiety (n=7), can view medications (n=7), proxy access to family member's chart (n=4), and previsit questionnaires (n=1).

^d^Secure messaging was done through MyChart and answered a question from the user.

^e^Other included visit more efficient because informed (n=9), health empowerment (n=8), and preferred to get results in person (n=3).

^f^Other included not paying for meals/hotels (n=10), not missing work (n=7), and not paying for childcare (n=2).

### Health System Usage

We studied the proportion of patients who had a no-show to an appointment, as it related to patient portal user status. From January 2016 to July 2018, there were 19,968 no-shows across the 5 clinics that participated. Of these visits, 9021 did not have an active patient portal account at the time of the visit. The number of no-shows amongst these visits for patients without a patient portal was 858 (9.5%, range: 3.7%-16.6%). Of the remaining 10,947 visits for patients with an active patient portal account, 493 (4.5%) no-show visits were recorded (range: 3.1%-10.4%). No-show rates for patients with no patient portal were 11.9% (386/3257) for the specialty clinics and 8.2% (472/5764) for the family practice clinic, whereas the rates for patients with patient portal access were 4.9% (194/3943) and 4.3% (299/7004), respectively. This represented a 53% relative reduction in the no-show rate overall (*P*<.001).

## Discussion

### Overview

In our study, patients were surveyed about their perceptions of an EMR-tethered patient portal. Patients had high general satisfaction, with over 90% reporting that it was easy to use, and almost half reporting that it saved them a medical visit. Respondents reported that access to results and advanced features, such as messaging and scheduling, improved communication with their care providers. Additionally, self-reported reductions in health system use implied improved utilization of scarce health system resources. Finally, objective data from clinic visits demonstrated an over 50% relative reduction in no-show rates.

We decided to focus on patient-centered indicators of patient portal utility rather than tracking health outcomes in our study. As with any technology, the benefits will accrue only if the technology itself is working and is being used as expected [24]. Although it is assumed that just having access to health information is of benefit, there is little evidence that access to lab tests will improve health outcomes. However, we believe that more advanced features, such as scheduling and secure messaging, in combination with access to test results and clinical notes are more likely to result in long-term health system benefits. In 2014, Goldzweig et al [17] performed a systematic review and found that there were a limited number of studies about patient portals, with heterogeneous designs making it difficult to draw strong conclusions about a new technology. Additionally, they found that improved outcomes (eg, for chronic disease such as diabetes, hypertension, and depression) tended to involve portal use in conjunction with case management [17]. They also found mixed data about the effect of portals on health care utilization and efficiency. Based on the success of the patient portal in our study, planning is underway to expand patient portal use across all AHS clinics. This expansion is targeted at the notion that patients having access to their own information in conjunction with the ability to interact with the health system digitally will contribute to increasing health system efficiency, including reductions in no-show rates and clinic and emergency department (ED) visits, increased patient satisfaction and empowerment and, in the longer term, improved health outcomes. The latter assumes that the patient portal is not only widely available, but actively being used as intended.

The 53% relative reduction in the no-show rate seen in patient portal users in the 5 pilot clinics may be widely relevant. Across the Edmonton zone of AHS, about 200,000 appointments are scheduled monthly**.** Of these**,** about 40,000 appointments are rescheduled, and 2000 appointments are considered no-shows. Patients who miss appointments often use emergency departments as sources of both primary and chronic care, driving up costs and straining hospital systems [23-25]. Missed appointments can compromise continuity and quality of care for both the patients who no-show and others who would have been scheduled in those appointment slots [25]. If the decreased no-show rates scale up to a wider set of users, patient portals may have significant positive impact on wait times and clinic efficiency. Access to scheduling functions in a patient portal may also be associated with decreased urgent care visits, although studies are not consistent [26,27]. In the current work, 2.7% of respondents reported being able to avoid one or more ED or urgent care visits, and 48% reported avoiding a clinic visit due to being able to communicate electronically. ED overcrowding is an ongoing and seemingly intractable problem; any strategy to safely decant these pressures in the Canadian context warrants further exploration.

In the near future, patients will expect web-based and mobile app–based access to their health data, and the ability to manage their health and interact with the health system digitally. In some jurisdictions, this access is not only related to tests and scheduling, but extends to transparent access into the complete medical record including progress notes [28]. Future studies will include interviews of care providers and patients to get a deeper understanding of the pros and cons of an EMR-tethered patient portal, its effect on clinic workflows, and the potential savings, whether that be in terms of time saved not waiting on hold during a phone call, or travel costs saved by replacing an inpatient visit with a secure message.

### Limitations

Our study evaluated a single patient portal in a large group of patients attending 1 of 5 clinics, in a single city, where the clinic leads were keen to implement it. It is not clear whether the results would be broadly generalizable. However, the study population did include both family medicine and specialty clinics, representing some diversity of needs and practice. Self-reported data is subject to recall bias, which we attempted to limit by sampling at different times after a user’s first successful patient portal access. The correlation between patient portal use and no-show rates may be partly the result of more diligent or engaged patients at the clinics participating in the study. Although we were able to compare survey respondent versus non-respondent demographic characteristics for all patients that had patient portal access, we did not have the ability to do the same for those with patient portal access versus those without. The analysis includes visits from non–patient portal users who eventually obtained patient portal access, which may underreport the effect size. The respondent population reported a high degree of comfort with computers, which may not hold true elsewhere, although it may reflect increasing competence with technology in general.

### Conclusions

Early experience with an EMR-tethered patient portal showed decreased no-show rates for appointments, high patient satisfaction, and self-reported changes in health system use. Implications on the expanded use of patient portals requires further study in Canada.

## Appendix

Multimedia Appendix 1 Survey Instrument.

## Abbreviations

AHS: Alberta Health Services
ED: emergency department
EMR: electronic medical record
