# Supplementary material for: Effects of a Web-Based Patient Portal on Patient Satisfaction and Missed Appointment Rates: Survey Study
Source: J Med Internet Res. 2020 May 19;22(5):e17955. doi: 10.2196/17955 (PMC7267992; doi:10.2196/17955)
Supplement: Multimedia Appendix 1 [file jmir_v22i5e17955_app1.pdf]

MyChart Online Survey

Online Survey

Using MyChart: How do you like it?

1. What do you use MyChart for? (Check all that apply)
    a. To access my health information
    b. To access my child(ren)'s health information
    c. To access my adult parent(s)' health information
    d. Other, please specify

2. What do you like most about using *MyChart*?
    a. It is easier to communicate with my family's care providers.
    b. It is easier to schedule appointments for myself and my family.
    c. I like being able to see my own lab results.
    d. I like to be able to view my own health information.
    e. I like to be able to view health information for people I care for, such as my family members.
    f. I like to be able to fill out clinical questionnaires in advance of my appointment.
    g. I like to be able to record my Blood Pressure for my care providers.
    h. I like to be able to record my glucose levels for my care providers.
    i. Other, please specify

3. Please indicate how much you agree with each of the following statements (Likert Scale, Strongly Agree to Strongly Disagree)
    a. MyChart is easy to use
    b. I think MyChart makes my care providers and clinic staff ask me to repeat myself less often
    c. MyChart saves me time when scheduling and appointment
    d. It is more convenient to communicate with my care providers using MyChart than using the method that I used to.
    e.
4. Do you find MyChart helpful?
    a. 0 = Completely unhelpful
    b. 100 = Completely helpful

5. Please tell us how we could improve MyChart (free text)

MyChart: Was it Helpful?

6. Do you think that using MyChart has helped you/your family avoid an Emergency Department or Urgent Care visit?

a. Yes
b. No
c. If yes please describe how you were able to avoid an Emergency Department or Urgent Care visit(s) as a result of using MyChart

7. Do you think that using MyChart has helped you/your family avoid a clinic visit(s) ?
    a. Yes
    b. No

8. If Yes, please explain how MyChart has helped you/your family avoid a clinic visit(s).
    a. Lab test or imaging results were discussed by secure messaging ("emailing") my care team.
    b. A question that I sent via MyChart secure messaging ("emailing") was answered in a way that avoided a visit.
    c. Other please explain

9. Please indicate which of these costs you saved on, by avoiding a clinic visit to your doctor.
    a. Gas
    b. Time off work
    c. Getting a babysitter
    d. Parking
    e. Taxi
    f. other, please specify

10. Where do you access MyChart from? (Choose all that apply)
    a. Desktop computer
    b. Mobile computer
    c. Smart phone
    d. Tablet device

11. My preferred way to access MyChart is
    a. Desktop or mobile computer
    b. Mobile phone
    c. Tablet device
    d. No preference

Your Health

Below are some statements that people sometimes make when they talk about their health. Please indicate how much you agree or disagree with each statement as it applies to you personally. Your answers should be what is <u>true for you</u> and not what you *think* others might want you to say. (Likert Scale: Disagree Strongly, Disagree, Agree, Agree Strongly, N/A)

12. When all is said and done, I am the person who is responsible for taking care of my health

13. Taking an active role in my own health care is the most important thing that affects my health
14. I am confident I can help prevent or reduce problems associated with my health
15. I know what each of my prescribed medications do
16. I am confident that I can tell whether I need to go to the doctor or whether I can take care of a health problem myself
17. I am confident that I can tell a doctor my concerns I have even when he or she does not ask
18. I am confident that I can follow through on medical treatments I may need to do at home
19. I understand my health problems and what causes them
20. I know what treatments are available for my health problems
21. I have been able to maintain (keep up with) lifestyle changes, like eating right or exercising
22. I know how to prevent problems with my health
23. I am confident I can figure out solutions when new problems arise with my health
24. I am confident that I can maintain lifestyle changes, like eating right and exercising, even during times of stress

Who are you?

25. What is your gender?
    a. Male
    b. Female
    c. Decline to Answer
    d. Other, please specify

26. What is your age range (in years)?
    a. <18
    b. 18-29
    c. 30-39
    d. 40-49
    e. 50-59
    f. 60-69
    g. 70 +

27. What is your level of comfort with computers?
    a. Completely comfortable
    b. Comfortable
    c. Neither comfortable nor uncomfortable (Neutral)
    d. Uncomfortable
    e. Completely uncomfortable

28. MyChart has the ability to connect your record to another person called a proxy. Please describe who you have connected proxy accounts to:
    a. My spouse

    b. My parent
    c. My child(ren)
    d. Other please describe.
29. Are you responsible for the care of family members?
    a. # of Children [slider with integer values]
    b. # of Adult Parents
    c. Others

Thank you very much for your time.
